# Hypersensitivity to intravenous ondansetron: a case report

**DOI:** 10.1186/1752-1947-2-274

**Published:** 2008-08-14

**Authors:** Karishma K Mehra, Nithya J Gogtay, Rohan Ainchwar, Lata S Bichile

**Affiliations:** 1 Department of Clinical Pharmacology, Seth GS Medical College & KEM Hospital, Parel, Mumbai, 400012, India; 2 Department of Medicine, Seth GS Medical College & KEM Hospital, Parel, Mumbai, 400012, India

## Abstract

**Introduction:**

Ondansetron, a 5-hydroxytryptamine_3 _receptor antagonist widely used in the prevention and treatment of chemotherapy-induced nausea and vomiting, is associated with various unusual adverse drug reactions. In this paper, we describe a hypersensitivity reaction to a single intravenous dose of ondansetron.

**Case presentation:**

A 19-year-old woman presented to the emergency department of our institute with 3–4 episodes of nausea, vomiting and epigastric distress. She had a diagnosis of polycystic ovarian disease and had been on treatment with cyproterone acetate 2 mg, ethinyl estradiol 0.035 mg, finasteride 5 mg and metformin 500 mg for a month. She had been taking oral roxithromycin 500 mg per day for the past 3 days for treatment of a mild upper respiratory tract infection. She also occasionally took rabeprazole 10 mg for gastritis which had worsened after treatment with roxithromycin. She was treated with a single 4 mg dose of ondansetron intravenously. She immediately developed urticaria, which was treated with intravenous dexamethasone 4 mg and chlorpheniramine maleate 20 mg. The reaction abated within a few minutes and she was discharged within an hour. She was asymptomatic at 72 hours of follow-up.

She had no history of ondansetron exposure, or drug or food allergies. On the Naranjo's causality assessment scale, the adverse event was 6 indicating a "probable" reaction to ondansetron.

**Conclusion:**

5-hydroxytryptamine_3 _receptor antagonists have been associated with life-threatening adverse reactions such as hypotension, seizures and anaphylaxis. The wide availability of these drugs in India has promoted their off label use in the treatment of gastritis, migraine and so on. Our case represents an off label use in a patient who could have been treated with a safer drug.

Some authors have suggested that anaphylaxis may be a class effect while others think it may be drug specific. In our case, the reaction could be either anaphylaxis or anaphylactoid, but the latter seems more likely given the history of absence of prior sensitization. Other components of the drug, such as solvent, also need to be considered as a cause of this reaction. Considering all of the existing evidence, we need to be more cautious while using ondansetron and also to be aware of the various unusual side effects, especially when used in an out-of-hospital set-up.

Our case report underscores the importance of physicians judiciously using the drug, particularly in the outpatient setting so as to reduce the incidence of avoidable adverse drug reactions.

## Introduction

Ondansetron is a 5-hydroxytryptamine_3 _(5-HT_3_) receptor antagonist widely used in the prevention and treatment of chemotherapy-induced nausea and vomiting, especially caused by highly emetogenic drugs such as cisplatin, and is considered a gold standard for this purpose [1]. It may also be used in the prevention and treatment of radiation induced nausea and vomiting as well as post-operative nausea and vomiting. Commonly seen side effects include constipation or diarrhea, headache and dizziness. All 5-HT_3 _receptor antagonists have been associated with asymptomatic electrocardiogram changes, such as prolongation of the PT and QTc intervals and certain arrhythmias [2]. The clinical significance of these side effects is unknown. Hypersensitivity to ondansetron is a rare side effect. In this paper, the authors describe a case of hypersensitivity to a single intravenous injection of ondansetron.

## Case presentation

A 19-year-old female patient visited the emergency department (ED) of a tertiary referral center with 3–4 episodes of nausea, vomiting and epigastric distress. She had been diagnosed with polycystic ovarian disease (PCOD) and had been on treatment with cyproterone acetate 2 mg, ethinyl estradiol 0.035 mg, finasteride 5 mg and metformin 500 mg for one month. The patient had also been taking oral roxithromycin 500 mg per day for the past 3 days for treatment of a mild upper respiratory tract infection. The patient also occasionally took a single dose rabeprazole 10 mg for gastritis. The gastritis had worsened after treatment with roxithromycin which was the cause of her visit to the ED. She was treated with a single 4 mg dose of ondansetron intravenously. Within a few seconds, the patient developed redness and wheals around the injection site along with urticaria. There was no hypotension or bronchospasm. She was immediately treated with intravenous dexamethasone 4 mg and chlorpheniramine maleate 20 mg. The reaction abated within a few minutes. The patient did not complain of any other symptoms and was discharged after an hour of observation. She was asymptomatic at 72 hours of follow-up.

On further history taking, the patient gave no previous history of use of ondansetron or other 5-HT_3 _antagonist exposure, and no drug or food allergies. There was no history of a similar episode in the past. She gave no personal or family history of atopy, asthma or bronchitis. On the Naranjo's causality assessment scale, the adverse event was 6 indicating a "probable" reaction to ondansetron [3].

## Discussion

5-HT_3 _receptor antagonists such as ondansetron, tropisetron, granisetron and palonosetron are generally associated with a wide safety margin and are widely used in cancer chemotherapy. There are, however, reports of life-threatening adverse events such as generalized tonic clonic seizures, hypotension [4], chest pain and dystonia [5]. To date, all anaphylaxis and anaphylactoid reactions induced by ondansetron have been in patients receiving the drug for cancer chemotherapy. This has prompted some authors to suggest that the drug's use should be restricted [6]. In the Indian market, the drugs have a wide availability with over 43 different brands. [2]. The wide availability of this class of drug has promoted the off label use of these drugs, such as in the treatment of antimalarial-induced vomiting, gastritis, migraines and other emetogenic conditions. The present case also represents the off label use of the drug in a patient who could have probably received safer medication such as domperidone or metoclopramide.

Some authors have suggested that anaphylaxis may be a class effect [7], while others think it may be drug specific [8]. Ondansetron and tropisetron share an indole heterocycle, while granisetron does not. This may justify the reports contradicting anaphylaxis as a class effect. While anaphylaxis is IgE mediated, anaphylactoid reactions are non-immune mediated. We did not determine IgE levels in this patient. A skin test was also not done, given the serious nature of the reaction. Thus the reaction could have been either anaphylaxis or anaphylactoid, but the latter seems more likely given the history of absence of prior sensitization. In 1993, Chen *et al. *reported that a total of 24 cases of varying manifestations of anaphylaxis or anaphylactoid reactions were reported to the United States Food and Drug Administration [9].

In the wake of the above evidence, and the increasing availability and off label use of ondansetron and other 5-HT_3 _receptor antagonists, we need to be more cautious while using this drug and also to be aware of the various unusual side effects, especially when used in an out-of-hospital set-up where prompt treatment of the reaction may not be possible. Our case report underscores the importance of physicians judiciously using the drug so as to reduce the incidence of similar avoidable adverse drug reactions.

## Conclusion

We emphasize the need to be judicious in the use of ondansetron and five other HT_3 _receptor antagonists due to their association with various unusual and life-threatening reactions. We also caution against the off label use of the drugs, especially in an out-of-hospital set-up.

## Consent

Written informed consent was obtained from the patient for publication of this case report. A copy of the written informed consent is available for review by the Editor-in-Chief of this journal.

## Competing interests

The authors declare that they have no competing interests.

## Authors' contributions

KM identified the adverse drug reaction and wrote the first draft of the paper. NG conceived the manuscript, performed the literature search, did the causality analysis and wrote the final draft of the paper. RA was the physician who treated the adverse drug reaction. LSB helped to draft and finalize the manuscript.
